# Esophageal intramural pseudodiverticulosis, a rare cause of food impaction: case report and review of the literature

**DOI:** 10.1093/gastro/gou035

**Published:** 2014-06-20

**Authors:** Yahuza Siba, Saritha Gorantla, Anand Gupta, Edward Lung, Joan Culpepper-Morgan

**Affiliations:** ^1^Department of Medicine, Columbia University Medical Center, Harlem Hospital Center, New York, NY, USA and ^2^Division of Gastroenterology, Department of Medicine, Columbia University Medical Center, Harlem Hospital Center, New York, NY, USA

**Keywords:** esophageal intramural pseudodiverticulosis, food impaction, dysphagia, esophageal candidiasis

## Abstract

Esophageal intramural pseudodiverticulosis (EIPD) is a rare, benign condition of uncertain etiology and pathogenesis, which usually presents with either progressive or intermittent dysphagia. Acute presentation with food impaction, requiring emergency esophago-gastroduodenoscopy (EGD), is rare. We report a case of EIPD presenting as food bolus impaction in an elderly black female. The patient had no previous history of dysphagia or odynophagia. Currently accepted risk factors, such as diabetes mellitus, chronic alcoholism, and reflux esophagitis, were not present in our patient. Emergency EGD established the diagnosis and also dislodged the food bolus. Histopathological evaluation of the mucosa diagnosed co-existent acute candidal infection. Medical treatment with proton pump inhibitor and azole antifungal led to resolution of her symptoms. Review of the literature revealed that stenosis, strictures, perforation, gastro-intestinal bleed, and fistula formation are potential complications of EIPD. Multiple motility abnormalities have been described but are not consistent. Treatment of the underlying inflammatory and or infectious condition is the mainstay of management of this unusual condition.

## INTRODUCTION

Esophageal intramural pseudodiverticulosis (EIPD) is a rare esophageal disease that was first reported in 1960 [1]. The etiopathogenesis of EIPD remains unknown; it has been associated with various risk factors, such as diabetes mellitus, chronic alcoholism, and reflux esophagitis. The main clinical symptom is chronic progressive or intermittent dysphagia. Although strictly a histological diagnosis, EGD or barium esophagogram have a characteristic diagnostic appearance. We report a case of EIPD in an elderly female, with no previous history of dysphagia, who presented acutely with food impaction.

## CASE PRESENTATION

**Figure 1:**
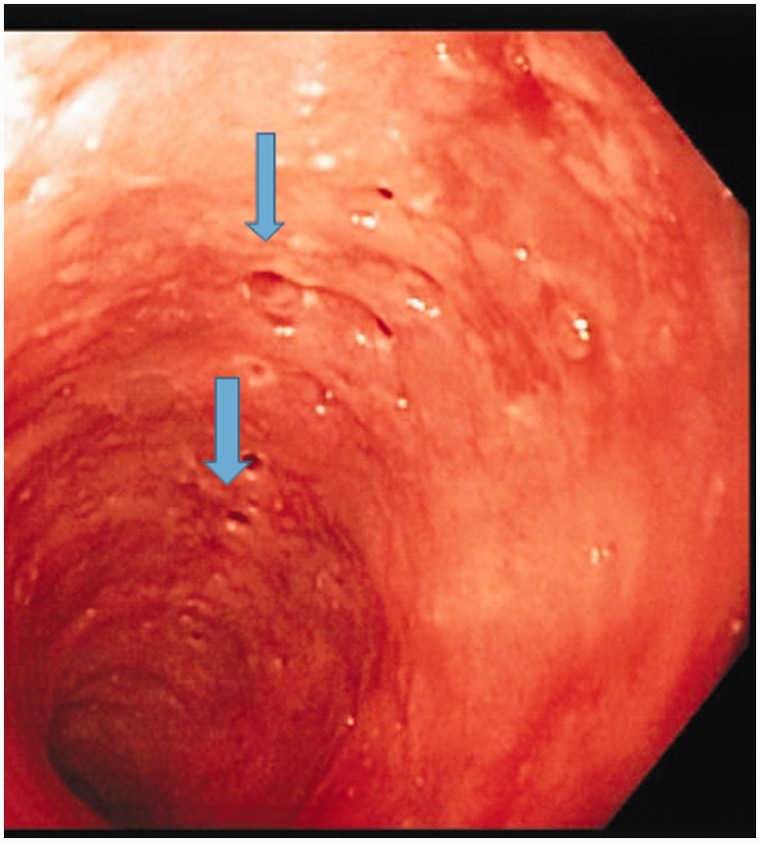
Multiple diffuse pseudodiverticula (thick blue arrows).

**Figure 2:**
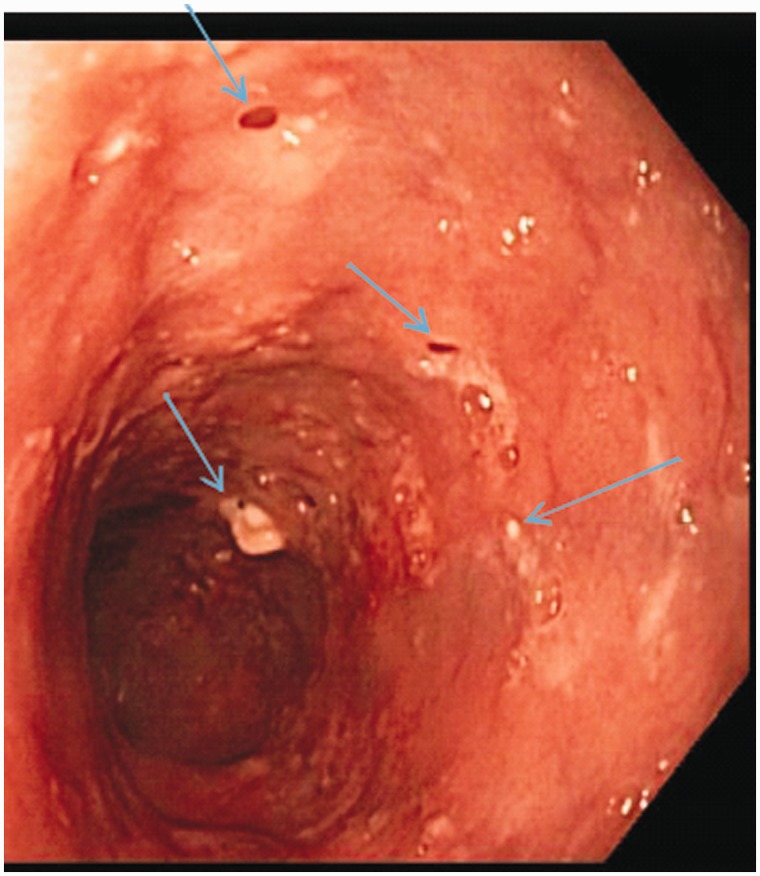
Numerous out-pouchings, some with cheesy secretions (light blue arrows).

A 78-year-old African-American woman, with past medical history significant for diet-controlled hypertension, was eating dinner at a local restaurant when she had a sensation of food stuck in her throat. Heimlich maneuvers were attempted, although the patient was able to breathe and talk. The emergency medical services (EMS) were called. She denied prior episodes of dysphagia, odynophagia, chest pain, weight loss, halitosis, corrosive ingestions, hematemesis or melena. She also denied any prior alcohol use and never smoked tobacco. Physical examination was unremarkable. Her complete blood count and basic metabolic panel were within normal limits. Emergency EGD revealed a food bolus in the mid-esophagus, which was easily pushed into the stomach. There was extensive erosive esophagitis in the distal esophagus. In addition, multiple small diverticulum-like openings were seen in the middle and proximal esophagus, and creamy mucoid discharge could be expressed from these openings by applying pressure. (Figures 1 and 2). Histology showed acute candida esophagitis; no evidence of malignancy or eosinophilic esophagitis. The patient was treated with fluconazole and a proton pump inhibitor (PPI), with resolution of her symptoms. One year after treatment she has not had any recurrence of symptoms and has declined surveillance EGD.

## DISCUSSION

Food bolus impaction is common. Causes include eosinophilic esophagitis, neoplasms, strictures, motility disorders, epiphrenic diverticula, esophageal webs, and rings. EIPD is an unusual cause of acute dysphagia; it is a rare, benign condition in which the wall of the esophagus develops numerous small out-pouchings. Mendl *et al.* first described the condition in 1960 [1]. Since then, sporadic cases have been reported; about 200 cases to date worldwide [2]. In a retrospective review of barium esophagograms, only 21 out of 14 350 (0.15%) had EIPD [3]. In an autopsy study, examinations from histological sections of esophagus taken randomly from 100 grossly ‘normal’ esophagi suggested that the early pathological changes of EIPD are more common than is appreciated. In that review, Medeiros *et al.* found that 67% had submucosal chronic inflammation associated with ducts or glands, 14% had dilated excretory ducts, and 7% developed cystic changes. They did not find any apparent relationship between submucosal chronic inflammation or duct dilatation and age [4].

On histology, EIPD is characterized by squamous, lined, dilated excretory ducts of deep mucous esophageal glands, often surrounded by chronic inflammation. Broadly speaking, these appear as pseudodiverticula. On EGD or barium study tiny (1–4 mm), flask-shaped out-pouchings are seen in longitudinal rows, parallel to the long axis of the esophagus. Bridging may sometimes occur between adjacent pseudodiverticula, resulting in distinct intramural tracking. The etiopathogenesis of EIPD is still undefined. It has been suggested that the pseudodiverticula are not the primary problem, but instead a consequence of secondary chronic esophagitis with periductal fibrosis, or due to esophageal hypermotility. This could result in compression or obstruction of squamous esophageal submucosal ducts or glands, resulting in formation of pseudodiverticula [2, 5].

Seventy-five percent of patients present clinically, with the remainder being diagnosed incidentally. In symptomatic patients, over 80% have presented with progressive or intermittent dysphagia, mainly due to strictures. Others present with symptoms resembling gastro-esophageal reflux disease (GERD), vomiting, chest pain, and chest tightness that may be misdiagnosed as cardio-pulmonary disease. Finally, some present with weight loss due to anorexia or, rarely, hematemesis or melena [2, 5, 6, 8]. Of the approximately 200 published cases of EIPD, none presented with a single episode of acute food impaction—as did our patient—without previous dysphagia or odynophagia.

Although EIPD can occur at any age—with the earliest reported case being an eight-month-old infant—it has bimodal peaks among teenagers and the 55–65-year-old age cohort; there is a slight male preponderance. Common underlying conditions are diabetes mellitus (approximately 20%) and chronic alcohol abuse (15%). Other risk factors are Crohn's disease, tuberculosis, Mallory-Weiss syndrome, achalasia, GERD or corrosive acid ingestions. Our patient did not have any of these risk factors; however, candida is a common finding, occurring in approximately 50% of patients, and it is unclear whether it is an incidental finding or has a role in etiopathogenesis (Table 1) [7, 9–12].

**Table 1:** Previously reported case reports/case series of EIPD showing author, year reported, clinical presentation, mode of diagnosis, etiology/comorbidities, and motility study findings

Author	Year	Clinical presentation	Mode of diagnosis	Comorbidities	Motility study
Mendl *et al.* [1]	1975	Dysphagia, weight loss, chest pain, throat discomfort	Barium	N/A	N/A
Sabanathan *et al.* [6]	1977	Dysphagia	Barium Negative EGD	Hiatal hernia (*n =* 9), sarcoidosis (*n =* 1), DM (*n =* 1)	High amplitude (*n =* 1), normal (*n =* 3)
Muhletaler [7]	1980	Dysphagia, vomiting, weight loss	EGD, barium	Strictures due to reflux (*n =* 3), lye ingestion (*n =* 2)	N/A
Hahne *et al.* [2]	1994	Dysphagia (*n =* 3), UGIB (*n =* 1), asymptomatic (*n =* 1)	EGD	Reflux esophagitis, candida esophagitis, DM, alcohol abuse	Decreased peristalsis (*n =* 1), normal (*n =* 2), non-specific (*n =* 1)
Upadhay *et al.* [11]	1996	Dysphagia	EGD, barium	Tuberculosis of esophagus	N/A
Yamamoto [12]	2001	Hematemesis	Barium	Mallory-Weiss	N/A
AtillaA, Maron NE [8]	2006	Food impaction	Repeat endoscopy	Alcohol abuse	N/A
Chon *et al.* [5]	2011	Chest tightness	EGD, barium	DM, hypertension, tobacco, alcohol	N/A
Halm *et al.* [13]	2014	Dysphagia	EGD	Alcohol (*n =* 23), tobacco (*n =* 23), alcoholic liver cirrhosis (*n =* 6)	N/A

DM = diabetes mellitus; EGD = esophago-gastroduodenoscopy; N/A = non-applicable; UGIB = upper GI bleed.

EIPD, although strictly a pathological diagnosis, is diagnosed clinically either by esophagogram or endoscopy. In 1985, in a review of 84 cases of EIPD, EGD only diagnosed 25% of cases detected by barium esophagogram; however, in a more recent study, Halm *et al.* (2014) reported that the sensitivity of both EGD and barium were similar, with EGD being the preferred method of establishing the diagnosis. The main reasons for missed diagnosis on EGD are lack of attention to the small ostia and the limited optical sensitivity of older instruments; however, with high definition endoscopes, the diagnosis is now less likely to be missed.

Classic EGD findings are numerous pits in the walls of the esophagus that are preferentially located proximally. Pseudodiverticula on barium examination may be segmental or diffuse in distribution. EIPD-associated strictures are also more common in proximal locations. Motility studies have shown various abnormalities, including aperistalsis, hypermotility or other non-specific findings. Our patient had no evidence of stricture; this is unusual compared with most reported cases, where the majority of patients had evidence of stricture on EGD or esophagogram [6, 13].

The mainstay of management of EIPD is treatment of the underlying or associated conditions and amelioration of symptoms. Due to association with GERD and esophagitis, PPIs are reasonable and commonly administered. Although the role of candida in etiopathogenesis remains elusive, antifungal therapy is usually prescribed in cases with documented candida. In one report a patient had severe stricture and stenosis that did not improve after repeated bougienage; however, dramatic symptomatic and endoscopic improvement was observed after oral azole therapy, suggesting anti-fungal therapy as a first-line treatment, even in patients with severe obstructive disease [14]. Our patient received both PPI and oral antifungal drugs. It is worth noting that EIPD treatment is not directed at the pseudodiverticula, as they rarely cause symptoms.

Although a benign disease, there are reports in which EIPD has been associated with esophageal cancer. Plavisc *et al.* suggested periodic surveillance for patients with EIPD, based on the finding of a higher prevalence of EIPD in patients with esophageal cancer, compared with controls (*P* < 0.001). Complications of EIPD include progressive stenosis, formation of strictures, bleeding, spontaneous perforation, broncho-esophageal and esophagomediastinal fistula, peridiverticulitis, pleural and pericardial effusion, and mediastinal abscess. Esophagectomy is sometimes required [15–16].

EIPD is a rare esophageal disease with unresolved etiopathogenesis, which can present with acute food bolus impaction (without antecedent esophageal disease) as well as with chronic progressive dysphagia. Endoscopy is invaluable in diagnosis and emergency treatment.

*Conflict of interest statement:* none declared.
